# Perturbation-based balance training to improve balance control and reduce falls in older adults – study protocol for a randomized controlled trial

**DOI:** 10.1186/s12877-020-01944-7

**Published:** 2021-01-06

**Authors:** Marissa H. G. Gerards, Rik G. J. Marcellis, Martijn Poeze, Antoine F. Lenssen, Kenneth Meijer, Rob A. de Bie

**Affiliations:** 1 Department of Physical therapy, Maastricht University Medical Center+ (MUMC+), Peter Debyelaan 25, 6229 HX Maastricht, the Netherlands; 2 Department of Epidemiology, Maastricht University, Peter Debyeplein 1, 6200 MD Maastricht, the Netherlands; 3 Care and Public Health Research Institute CAPHRI, Maastricht, The Netherlands; 4 Department of Surgery, division of Trauma surgery, MUMC+, Peter Debyelaan 25, 6229 HX Maastricht, the Netherlands; 5 School of Nutrition and Translational Research in Metabolism NUTRIM, Maastricht, The Netherlands; 6 Department of Movement Sciences, Maastricht University, Universiteitssingel 50, 6229 ER Maastricht, the Netherlands

**Keywords:** Accidental falls, Aged, Balance, Perturbation, Prevention, Older adults

## Abstract

**Background:**

Falls are a common cause of injuries and hospitalization among older adults. While conventional balance training appears effective in preventing falls, a relatively large number of training sessions are needed and retention of the effects after the training period is hard to accomplish. This may be because these interventions are not sufficiently task-specific for the mechanism of falls. Many falls in older adults occur due to unexpected external perturbations during gait, such as trips. Therefore, there is increasing interest in perturbation-based balance training (PBT), which is a more task-specific intervention to improve reactive balance control after unexpected perturbations. The literature suggests that PBT may be more effective and require fewer training sessions to reduce falls incidence in older adults, than conventional balance training. We aim to evaluate the effect of a three-session PBT protocol on balance control, daily life falls and fear of falling. Secondly, we will evaluate the acceptability of the PBT protocol.

**Methods:**

This is a mixed-methods study combining a single-blind (outcome assessor) randomized controlled trial (RCT) using a parallel-group design, and qualitative research evaluating the acceptability of the intervention. The study sample consists of community-dwelling older adults aged 65 years and older who have recently fallen and visited the MUMC+ outpatient clinic. Subjects are randomized into two groups. The control group (*n* = 40) receives usual care, meaning referral to a physical therapist. The intervention group (*n* = 40) receives usual care plus three 30-min sessions of PBT in the Computer Assisted Rehabilitation Environment. Subjects’ balance control (Mini-BESTest) and fear of falling (FES-I) will be assessed at baseline, and 4 weeks and 3 months post-baseline. Daily life falls will be recorded with falls calendars until 6 months after the first follow-up measurement, long-term injurious falls will be recorded at 2-years’ follow-up via the electronic patient record. Acceptability of the PBT protocol will be evaluated with semi-structured interviews in a subsample from the intervention group.

**Discussion:**

This study will contribute to the evidence for the effectiveness of PBT using a training protocol based on the available literature, and also give much needed insights into the acceptability of PBT for older adults.

**Trial registration:**

Nederlands Trial Register NL7680. Registered 17-04-2019 – retrospectively registered.

**Supplementary Information:**

The online version contains supplementary material available at 10.1186/s12877-020-01944-7.

## Background

Falls are a common cause of injuries and hospitalization among older adults [1]. Each year, one in three adults aged 65 years or older, and one in two adults above the age of 80 years, experience a fall [2]. In the Netherlands, around 108,000 older adults (332 per 10.000) visited the emergency department in 2018 after a fall incident, and 33% of them were subsequently admitted to hospital [3]. Falls are putting increasing demands on healthcare resources, with fall-related medical costs in the Netherlands of about 960 million euros in 2018 [3]. If the incidence of falls remains unchanged, and with the aging of the population expected to increase, the number of falls in adults aged 65 years and older will have increased by 47% by 2050 [3]. Falls often have serious physical consequences, such as fractures or head injuries [4]. In addition, there are psychological consequences of falling, which can have a strong negative impact on quality of life [5]. Up to 73% of older adults who have experienced a fall are afraid of falling again, which in turn can lead to decreased physical and social mobility [6]. Once an older adult has experienced a fall incident, their risk of sustaining future falls is greatly increased (OR 2.8 for all fallers, and 3.5 for recurrent fallers) [5, 7].

A modifiable risk factor that has repeatedly been identified in the literature is the presence of gait or balance problems [2, 5, 8]. Many studies have shown that balance training can effectively reduce falls incidence in older adults, with or without specific disorders, by approximately 24% [9–14]. However, the optimal type, duration and frequency of balance training to reduce falls are not yet clear. Berg et al. described three aspects of balance, which should be functionally adequate to accomplish functional balance [15]. The first is the ability to maintain various postures, also referred to as static balance control. The second, is the ability to make postural responses to voluntary changes of body position, using mostly proactive balance control. The third is the ability to react to unexpected external disturbances (perturbations) of balance, also called reactive balance control [15].

Conventional balance training has mostly focused on the first two aspects of balance, where proactive mechanisms of balance control are the most important [16]. While conventional balance training interventions appear effective in preventing falls, a relatively large number of training sessions are needed and retention of the effects after the training period is hard to accomplish [17–19]. This may be because many falls in older adults occur as a result of an unexpected external perturbation during gait, such as a slip or a trip [20, 21]. The unexpected nature of such external perturbations forces individuals to rely mostly on reactive balance control. Since balance training seems highly task-specific, it is not likely that training proactive balance control will also improve reactive balance control, in view of the additional speed and stability requirements of these balance reactions [22].

During the process of physiological aging, changes in the body lead to less efficient reactive balance control strategies, such as delayed onset of muscle responses, decreased magnitude of postural responses, and an increased level of co-activation in muscles [23–25]. Even in community-dwelling older adults who walk independently, there may be a substantial decline in reactive balance control, but this will not become evident until a slip or a trip occurs [26]. Despite this decline, the potential to adapt and improve reactive balance control through training seems to be retained with age [27], leading to an increasing interest in perturbation-based balance training (PBT) [28].

PBT is a form of training that aims to improve reactive balance control after unexpected external perturbations. In a safe and controlled environment, participants are repeatedly exposed to destabilizing perturbations during various activities of daily living. Many different training setups can be utilized, from fairly simple lean-and-release perturbations requiring only a safety harness, to advanced systems that can provide a wide variety of perturbation types and intensities during various tasks.

Studies of PBT have shown significant reductions in falls in older adults with and without specific disorders such as Parkinson’s disease or stroke (with a relative risk of falls of 0.71 [95% CI 0.52–0.96] compared to various control groups) [29–36]. Adaptation may occur faster with this type of training than with conventional balance training, offering the potential of achieving equal or better results with fewer training sessions [34]. For example, a study by Pai et al. showed a 50% reduction in the incidence of daily-life falls during 12 months of follow-up after only a single training session [34].

In an earlier review, we included eight studies on PBT in older adults [28]. We concluded that PBT appears to be a feasible approach to falls prevention in clinical practice, and that a combination of types and directions of perturbation might offer the greatest benefits. Frequency and volume of training varies greatly between studies, and while there are studies showing positive effects with relatively low training doses, the optimal training characteristics are not yet clear. In this study protocol, we describe a PBT protocol including multiple types and directions of perturbations, with a training dose that we hypothesize to be suitable based on the current evidence [28].

Besides improving balance and reducing falls incidence, PBT can significantly reduce the fear of falling [37]. Fear of falling can have a major impact on older adults. While it may initially be a reasonable response to experiencing a fall, and may lead to more cautious behavior, it can also have debilitating consequences when it leads to activity restriction [6]. If this occurs, fear of falling can lead to physical deconditioning and frailty, which can set off a negative spiral by increasing the risk of recurrent falls [38].

While the body of evidence for the effectiveness of PBT for falls prevention is growing, there are other factors to consider [8, 32, 34–36, 39–41]. The perturbations applied in these studies are of such a magnitude that they may not be acceptable to all older adults. Even the most effective interventions are likely to fail if they are not acceptable to the target population. Therefore, the literature recommends combining quantitative and qualitative methods to assess both the effectiveness and acceptability of a new intervention [42]. In this study, we will assess the acceptability of our training protocol through semi-structured interviews, utilizing the definition and theoretical framework of acceptability (TFA) described by Sekhon et al. [43].

This study protocol describes a mixed-methods study combining an outcome-assessor blinded randomized controlled trial (RCT), using two parallel groups (1:1) and a superiority design, with qualitative research concerning the acceptability of the intervention. The primary aim is to determine the effect of our three-session PBT intervention on balance control measured with the Mini-BESTest in community-dwelling older adults (≥65 years) who visit the outpatient clinic after a fall, in comparison to usual care. Secondly, we aim to determine the effect on real-life falls incidence during a 6 months follow-up period. We will also evaluate the effect on fear of falling measured with the Falls Efficacy Scale International (FES-I). A long-term follow-up evaluation will take place at 2 years post-baseline when each subjects’ electronic patient record (EPR) will be checked with the aim of investigating the long-term effect of PBT on injurious falls.

Lastly, we aim to determine the acceptability of our three-session PBT protocol through semi-structured qualitative interviews in a sub-group of the intervention group in this study.

## Methods

### Study design

This is a mixed-methods study combining a single-blind (outcome assessor) randomized controlled trial (RCT) in a parallel-group design, with qualitative research to assess the acceptability of the intervention. This protocol was written in accordance with the Standard Protocol Items: Recommendations for Interventional Trials (SPIRIT) checklist. The study will be conducted at MUMC+ in Maastricht, the Netherlands from March 2019 until July 2021. The study was approved by the Medical Ethics Committee azM/UM (METC18–049).

### Subjects

Community-dwelling older adults (65+) who have experienced a fall in the past 3 months and visit the MUMC+ outpatient clinic will be approached to participate in this study. To ensure that our sample includes only older adults at increased risk for falling, persons who fell during exercise activities (i.e. cycling) or due to actions of a third party, will be excluded. Similarly, persons using medication that is known to increase the fall risk will be excluded from this study. For a full description of the eligibility criteria, see Table 1.

**Table 1:** Full eligibility criteria

Inclusion criteria	Exclusion criteria
Age ≥ 65 years	Diagnosed with osteoporosis
Community-dwelling	Recent fracture or severe contusion of the lower extremities, back or shoulders (in consultation with medical doctor)
Able to walk without a walking aid for ≥15 min	Any disease or disorder that may influence the safety of training (e.g. severe cardiopulmonary disease)
Recently having experienced a fall (≤3 months ago)	Falls caused by actions of third parties or during exercise activities
Having visited the MUMC+ outpatient clinic after their fall incident	Uncorrected vision problems
	Falls due to syncope
	Use of medication known to increase fall risk (antidepressants, benzodiazepines, sedatives, hypnotics, antipsychotics) [44]
	Use of painkillers that can decrease responsiveness (e.g. morphine, oxycodone) [44]
	Inability to provide written informed consent and communicate in Dutch
	Inability to follow instructions due to cognitive problems

### Recruitment, randomization, blinding and treatment allocation

Eligible patients will be informed about this study by their medical doctor when they visit the outpatient clinic of the MUMC+. If patients are interested in the study, they will receive written information and will be asked for their permission to be phoned by the investigators. Patients are given the opportunity to read the study information at home and will be phoned by the investigators 3 to 7 days later. If a patient wants to participate, an appointment will be scheduled to visit the MUMC+ department of physical therapy. During this visit, any remaining questions will be answered and written informed consent will be obtained.

After this, the investigator will check the eligibility criteria. If the subject meets the criteria, baseline measurements will be performed. When these have been completed, the subjects will be randomized to the intervention- or control group. This will be done using a 1:1 ratio stratified block randomization (block sizes 2 and 4 will be randomized). Randomization will be stratified based on sex (male versus female) and number of falls during the past year (1 versus 2 or more). This stratification will result in four different strata. The randomization sequence will be generated using an online random number generator. The allocation will be concealed by using sequentially numbered, sealed opaque envelopes. The person preparing the allocation concealment mechanism will be a different person than the one enrolling the subjects and assigning them to a group. The allocation sequence list will be kept in a locked drawer, which can be accessed only by the principal investigator.

### Timeline

The first study visit for subjects consists of two parts; t_− 1_ is where subjects make their final decision on participating in the study, informed consent is obtained and the eligibility criteria are checked. If a subject meets all eligibility criteria, this visit is combined with t_0_. At t_0_, baseline measurements are performed by the outcome assessor, which will be blinded to treatment allocation. Subjects are explicitly instructed to hide their treatment allocation from this researcher. If the treatment allocation of an individual subject is revealed to the outcome assessor, a second blinded outcome assessor will take over the remaining measurements. After baseline measurements, the subject is randomized to the control- or intervention group by the same researcher that enrolled the subject in the study. For subjects in the intervention group, this visit is followed by three training sessions in 3 weeks. Both groups have their t_1_ visit at 4 weeks post-baseline, and a t_2_ visit 3 months after that, t_2_ being the last study visit for subjects. From t_0_ on, all subjects will fill in falls calendars until 6 months after t_1_. A final check of the EPR at t_3_, 2 years post baseline, concludes data collection for this study. An overview of the study timeline is presented in Table 2.

**Table 2:**
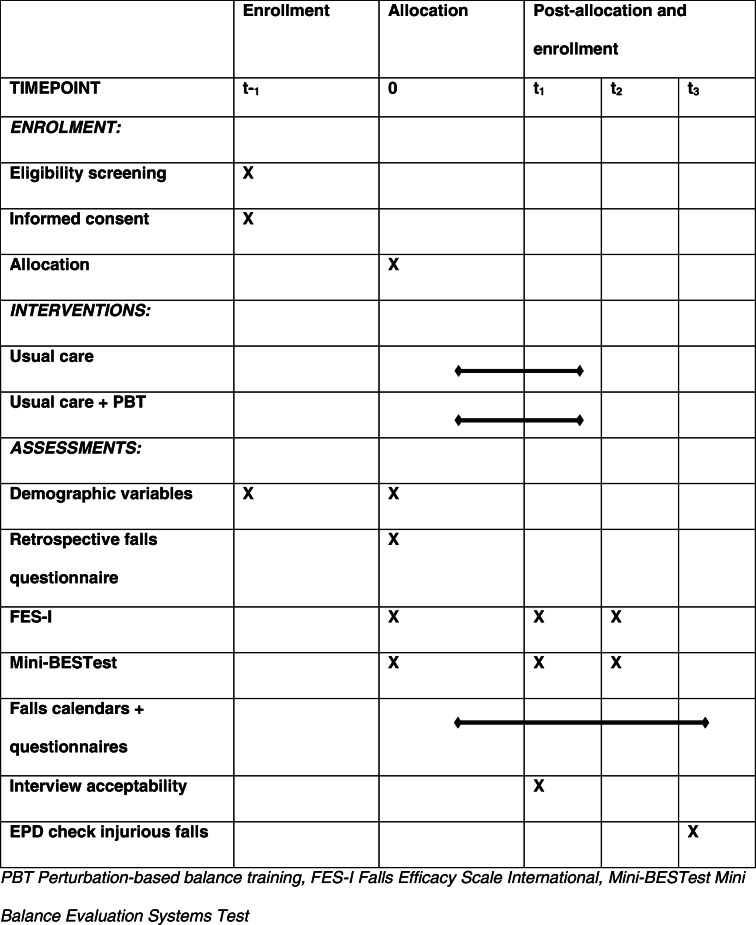
Patient flow of enrolment, assessments and interventions

### Interventions

Subjects in this study will be randomized to the control group (usual care) or the intervention group (usual care + PBT). Training in the intervention group will be provided by specifically trained physical therapists in association with clinical operators of the CAREN system.

### Control intervention (usual care)

All included subjects will receive usual care. Usual care in the MUMC+ outpatient clinic consists of a referral for physical therapy treatment for the injuries sustained during the fall incident, if the medical doctor determines that this is indicated (for example, mobility and strength exercises after a shoulder fracture). During the study, the outcome assessor will monitor whether each subject has used their referral to visit a physical therapist, how many times, and what components (i.e. strength training, mobility exercises, balance exercises) were included in the physical therapy treatment.

### Experimental intervention

The experimental intervention in this study is PBT. The aim of this training program is to improve reactive balance control in older adults by practicing balance recovery from unexpected perturbations in a safe and controlled environment.

#### Training setup

Training will take place on the CAREN system at the MUMC+ department of physical therapy. The CAREN is a dual-belt treadmill system embedded in a motion platform with 6 degrees of freedom and surrounded by a 180 degree screen. The treadmill and the motion platform can both provide reactive balance challenges separately or combined, providing a wide array of possible types and directions of perturbation. Virtual reality environments are projected onto the screen to make the training activities more immersive. Subjects will wear a safety harness at all times during training to protect them from injuries in case of an unsuccessful balance recovery.

#### Training dose

Based on previous studies, we hypothesize that three training sessions of 30 min each will be enough to facilitate adaptation of reactive balance control [32, 34–36, 39–41].

#### Perturbation intensity and progression

While it is not clear exactly how perturbation magnitude impacts motor learning and retention, it appears that high-magnitude perturbations (where the subject initially needs the safety harness to recover their balance) result in fast and significant adaptation with long-term skill retention [28]. However, with regard to the safety and acceptability of training, a more progressive and personalized approach that is still challenging seems more reasonable. We therefore decided to monitor how challenging the training is for each subject, and to individualize the progression of difficulty levels. With this goal in mind, we will use a numeric rating scale (NRS) where each subject will rate the difficulty of maintaining balance control during training on a scale from 1 to 10. The following guideline will be used to interpret how challenging the training is for each subject; NRS 1–3 barely challenging, NRS 4–5 mildly challenging, NRS 6–7 challenging, NRS 8–9 very challenging, NRS 10 unsuccessful balance recovery. The NRS scale will be monitored regularly during training, with the aim to train at an NRS of 6 to 9. If a subject scores the level of challenge as below 6 and this score is consistent with the subjective impression of the physical therapist, the perturbation difficulty level will be increased. The maximum perturbation difficulty levels displayed in Tables 3, 4, 5 and 6 are based on the possibilities of the CAREN system, and on pilot testing with healthy older adults.

**Table 3:** Perceived difficulty scores, according to the NRS score and their corresponding increase in difficulty level

NRS score (0–10)	Increase in difficulty level (percentage)
1	15%
2	15%
3	15%
4	10%
5	10%
6	5%
7	5%
8	0%
9	0%
10	0% or decrease by 5–10%

*NRS score* Numeric Rating Scale score. Increase in difficulty level: The percentage by which the difficulty level will be increased if the subject scores the corresponding NRS score

**Table 4:** Difficulty levels and their corresponding perturbations of platform displacement and maximum speed of shift

Difficulty level	Displacement (cm)	Maximum speed (m/s)
1	5	0.11
2	7.5	0.16
3	10	0.21
4	12.5	0.26
5	15	0.31
6	17.5	0.36
7	20	0.41

Displacement: The distance in centimeters which the platform will move during a perturbation of a certain level. Maximum speed: The maximum speed at which the corresponding platform displacement will be reached

**Table 5:** Difficulty levels and their corresponding perturbations of platform angles and maximum speed of the platform tilt

Difficulty level	Tilt left/right (degrees)	Tilt forward/backward (degrees)	Speed (degrees/s)
1	3	2	6.2
2	4.5	3.5	9.2
3	6	5	12.2
4	7.5	6.5	15.2
5	9	8	18.2
6	10.5	9.5	21.2
7	12	11	24.2

Tilt: The number of degrees by which the platform will tilt to a certain side during a perturbation of the corresponding level. Speed: The maximum speed at which the corresponding platform tilt will be reached

**Table 6:** Difficulty levels and their corresponding perturbations of treadmill belt acceleration/deceleration, speed (increase) and duration of unilateral treadmill acceleration and deceleration

Difficulty level	Acceleration/Deceleration (m/s^2^)	Speed (increase/decrease, m/s)	Duration (s)
1	3	0.5	0.20
2	3	0.85	0.28
3	3	1.2	0.36
4	3	1.55	0.44
5	3	1.9	0.52
6	3	2.25	0.60
7	3	2.5	0.68

Speed: The increase or decrease in unilateral treadmill belt speed in m/s for each difficulty level. Duration: The amount of time during which the increased speed is maintained

#### Perturbation types

From the available literature, we concluded that incorporating multiple perturbation types and directions might be of most benefit [28]. Each training session will therefore incorporate platform (translations and tilts in the forward, backward, left and right direction) and treadmill perturbations (unilateral treadmill belt accelerations and decelerations) during standing and walking. Each perturbation type has seven increasing difficulty levels (Tables 4, 5 and 6). For each subject, the first training session will be started with perturbations of each type on level 1. Difficulty levels will then be increased based on individual training progression and NRS scores.

#### Training procedures

The first training session on the CAREN system will start with a period of familiarization, where the subject can get used to the system by walking on the treadmill. Subjects will report an NRS score for how comfortable they feel walking on the CAREN before and during familiarization (0; very uncomfortable to 10; fully comfortable). If a score of 7 or higher is reached, familiarization is complete. We expect that this will occur within 6–7 min [45].

After this, each subjects’ comfortable walking speed will be determined using a ramped protocol; the walking speed will start at 0.5 m/s and will gradually be increased until the subject says ‘stop’ when they think their comfortable walking speed is reached. The subject will walk at this speed for approximately 1 min to check if any adjustments need to be made.

The consecutive sessions will start with a warming-up during which the subject will walk on the treadmill for approximately 3 min on a level surface and readjust to the system. Each training session will consist of three parts: gait adaptability, static reactive balance, and dynamic reactive balance. Training difficulty can be progressed by increasing the perturbation magnitude and walking speed. During the second and final training sessions, cognitive and motor dual tasks can also be added to increase training difficulty. Training adherence will be monitored throughout the study, and subjects will be encouraged to reschedule any missed training sessions.

#### Gait adaptability

Subjects will walk in a virtual environment on a path through a forest, with various slopes and turns. Both the incline/decline of the slopes and the sharpness of the turns will have a standardized starting level of 20% (out of 100%), which will then be progressed in steps of 5 to 15%. A difficulty level of 20% corresponds to a maximum incline/decline and rotation of 2 degrees. Each 5% increase in difficulty level means an increase in the maximum incline, decline and rotation of 0.5 degrees. For the percentage by which the difficulty level will be increased for each NRS score, see Table 3.

#### Static reactive balance

Subjects will stand on the CAREN while the platform and treadmill make sudden movements. The platform can shift or tilt to anterior, posterior, left and right. The treadmill belt can unilaterally accelerate from standstill. All possible perturbations have seven difficulty levels (see Tables 4, 5 and 6). Training will start at level 1 for each subject.

#### Dynamic reactive balance

Subjects will walk on the treadmill at their comfortable walking speed, while the above mentioned platform and treadmill perturbations are applied. The treadmill perturbations will consist of unilaterally accelerating or decelerating the treadmill belt for short periods of time, simulating a trip or a slip, respectively (Table 6).

### Outcomes

#### Balance control (main outcome)

The main outcome in this study is balance control, which will be measured with the Mini Balance Evaluation Systems Test (Mini-BESTest). The Mini-BESTest has been identified as the most comprehensive measurement tool to assess balance in community-dwelling older adults [46]. It measures balance in four categories: proactive balance, reactive balance, sensory orientation and dynamic gait. Each of the 14 tasks is scored on a three-point scale, with a total score that can range from 0 to 28 points. A higher score corresponds with better balance control. Proactive balance is tested using a sit-to-stand transfer where the subject has to try not to use their hands, standing on tiptoes, and standing on one leg. Reactive balance is tested with therapist-applied lean-and-release perturbations in the forward, backward and sideways directions. Sensory orientation is tested by standing with feet together, standing with feet together and eyes closed on a foam surface, and standing on a slope with eyes closed. Dynamic gait is tested with five tasks; suddenly changing gait speed, walking while turning the head left and right, walking and turning, stepping over an obstacle while walking, and Timed Up and Go performance. The Mini-BESTest has good psychometric properties [47, 48]. Cut-off values indicating increased fall risk for the Mini-BESTest are age dependent; ≤25 points for people 60 to 69 year of age, ≤23 points for 70 to 79 years, ≤22 points for 80 to 89 years and ≤ 17 points for 90 years and older [49]. The minimal detectable change on the Mini-BESTest is 3.5 points and the minimal important change is 4 points [47]. Balance control will be measured at baseline, T_1_ and T_2_.

#### Retrospective falls incidence

At baseline, the number and circumstances of falls during the past year of each subject will be recorded. A fall is defined as ‘an event which results in a person coming to rest inadvertently on the ground or floor or lower level’ [50]. We will use an adapted version of the ‘falls history questionnaire’ as presented in the book *‘Falls in older people: risk factors and strategies for prevention’* [51]. This questionnaire records if a fall has occurred in the previous period (12 months), where it has happened, what the perceived cause was, and if and what kind of injuries were sustained. The outcome assessor will fill in this questionnaire together with the subject to make sure that the recorded data is as comprehensive and clear as possible.

#### Prospective falls incidence

From the moment of inclusion, the prospective falls incidence of each subject will be monitored for up to 8 months (the potential training period + 6 months) post baseline. The prospective falls incidence will be monitored with falls diaries and questionnaires. The falls diary is a calendar that the subject will fill in at the end of each day. They are instructed to put an ‘X’ if they did not fall that day, or a number representing the number of times they fell during that day. At the end of each month, subjects will fill in the falls history questionnaire about that particular month, and send this back to the researchers in pre-addressed and pre-paid envelopes. If a fall incident is reported, the researcher will follow this up with a short phone call to elaborate on the circumstances and consequences of this fall. If a calendar has not been returned within 10 working days after the end of the month, the researcher will remind the subject with a phone call. A final follow-up will take place at 2 years post-baseline, where the researcher will check each patients’ EPR, to see if there were any more hospital visits due to injurious falls.

#### Fear of falling

Fear of falling will be measured with the Falls Efficacy Scale International (FES-I, Dutch version). This version of the falls efficacy scale is a 16-item questionnaire developed to determine if a person has confidence in their ability to perform a range of daily activities without falling. It has been adapted to be more suited to older adults, including a range of activities from very basic to more complex [52]. The questionnaire will be filled in by the subject with the help of the outcome assessor. Sixteen items are scored on a four-point [1–4] scale, with a maximum score of 64 points. A higher score corresponds with a greater fear of falling. The Dutch version of the FES-I has good reliability and validity and discriminative power in older adults [52–55]. Fear of falling will be measured at baseline, T_1_ and T_2_.

#### Acceptability of the intervention

This study will evaluate not only the effect but also the acceptability of the intervention. In a sub-sample of the intervention group, semi-structured interviews will be conducted to investigate the acceptability of the PBT protocol. Each of these subjects will be interviewed once (for approximately 30 min) after completing the intervention. The interview guide will be based on the TFA, which consists of seven subsections:

- Affective attitude: how does the subject feel about the intervention, what is their opinion about it?
- Burden: did participating in the intervention take (a lot of) effort in terms of exertion or balance?
- Ethicality: did the intervention fit the subject’s previous views on falls prevention?
- Intervention coherence: did the subject understand the goal(s) of the intervention and how it works?
- Opportunity costs: did the subject have to give up other opportunities to take part in the intervention (e.g. cancel other appointments)?
- Perceived effectiveness: did the subject notice any effects (physical or otherwise) of the intervention, during training or after their participation?
- Self-efficacy: how confident was the subject about their ability to participate in and complete the intervention?

Two interviewers will be present at each interview; one will lead the interview, while the other will observe non-verbal communication, make notes, and help keep track of the interview guide. In addition to the framework, any perceived barriers/facilitators to participating in the intervention will be discussed. The interviews will take place at MUMC+ and will be administered by the research staff who are familiar with the training protocol.

### Data management

Each subject will receive a unique identification code when they are included. All data will be collected and stored anonymously and linked to this code. Data will be collected on a paper case report form and paper questionnaires. The data will then be digitized by a trained research assistant and will be verified by the coordinating researcher before analysis. Trial conduct and a sample of the data will also be verified by an independent research monitor during and at the end of the study, and at two time-points during the study. Data from the semi-structured interviews will be recorded using a voice recorder and saved in a password-protected folder, while regular back-ups will be made to a password-protected external hard drive. The recordings will be deleted from the voice recorder immediately after they have been saved on the computer and external hard-drive.

### Sample size

Sample size was calculated in G*power 3.1.9.2 and was based on the primary outcome of this study, difference between means on balance measured with the Mini-BESTest at T_1_. The effect size *d* (0.61) was calculated based on values from an article with a similar study population and an intervention aimed at improving dynamic balance control [56]. We assumed that our control group would remain stable as they receive no extra training aimed at balance improvement. Sample size was based on a one-tailed independent samples t-test, an α of 0.05, power (1-β) of 0.80 and allocation ratio of 1. This results in a required sample size of *n* = 72. Accounting for an expected loss to follow-up of 10%, the final sample size is *n* = 80. Sample size was estimated conservatively, making no assumptions about the correlation between predictors (group allocation and baseline score) added into the model and the outcome variable.

For the purpose of investigating the acceptability of the PBT protocol, a sub-sample from the intervention group will be included. The sample size will be based on the concept of theoretical saturation as described by Glaser & Strauss [57]. If there are two consecutive interviews that provide no new information, no new interviews will be planned. We expect to include approximately 15 subjects for the interviews.

### Statistical analysis

Data will be analyzed using the Statistical Package for the Social Sciences (SPSS) version 23.0 (SPSS, Chicago, Ill., USA). Descriptive statistics will be used to explore the data and will be presented in tables and figures. Data will be displayed as mean and standard deviation (SD) or as median and interquartile range (IQR), depending on normal distribution of the data. Categorical data will be summarized by frequency (n) or percentage (%). Data will be analyzed on an intention-to-treat basis; missing data will be imputed using multiple imputation. In all statistical analyses, statistical significance will be set at *p* < 0.05. Data-analysis will be performed by a researcher who is blinded to the group allocation.

Primary analysis will test whether there is a significant difference in balance, as measured with the Mini-BESTest, between the control- and intervention groups at T_1_. This will be analyzed using multiple linear regression. Based on the randomization, no between-group differences in variables are expected. However, if there are differences at baseline (based on qualitative appraisal of the baseline table), these factors will be entered into the model. The secondary study parameter ‘fear of falling’ will be analyzed using the same method. The incidence of falls will be analyzed using generalized linear regression with a link function appropriate for the number of falls, using the mean number of retrospective and prospective falls per person-time unit.

A longitudinal analysis will be performed as a secondary, more explorative analysis of balance (Mini-BESTest) and fear of falling (FES-I) over time. A linear mixed effect model will be used to assess the treatment effects, since this model uses all available data, accounts for missing data using a likelihood based method - where missingness at random (MAR) is assumed - and takes the correlation between repeated measurements within a subject into account. The fixed part of the model consists of time, time*treatment, and other covariates that are related to the outcome (power gain) and/or to the missingness (to ensure MAR). The treatment factor, indicating the different treatment groups, is not included, since no group effect is assumed at baseline due to randomization. As for the random part of the model, an unstructured covariance structure is assumed for the repeated measures.

For the analysis of the qualitative data, the interviews will be transcribed verbatim. All transcriptions will be double-checked by a second researcher. A summary of the interview will be sent to the subject, so they have the opportunity to check the information and provide feedback. A deductive content analysis based on the theoretical framework of acceptability will be performed on the data using NVivo software, version 12 (QRS International). The transcripts will be coded independently by two researchers. After this, any differences in coding will be discussed. A third researcher will be available to reach consensus if necessary. An unstructured categorization matrix will be utilized, so topics that do not fit the initial framework can be added based on inductive content analysis [58]. If new topics arise during data collection, they will be added to the interview guide.

Circumstances and consequences of fall incidents, whether injurious or not, will be reported qualitatively, and using descriptive statistics.

### Adverse events

All adverse events until 2 weeks post T_1_, and after that all spontaneously reported adverse events, will be recorded and reported in the final publication of this study.

### Trial status

Enrolment for this study started on March 12, 2019. Recruitment for this study is currently ongoing, and is expected to be completed by December 31, 2020.

## Discussion

### Strengths

This study will assess the effects and acceptability of a PBT intervention compared to usual care in an outpatient setting. It is designed as a mixed-methods randomized controlled trial in which, not only the effectiveness, but also the acceptability of the intervention will be assessed, to facilitate further development and implementation in clinical practice of this potentially effective intervention. The training protocol for the intervention has been developed based on principles of training and a critical review of findings from the available literature. It is described in detail to improve reproducibility, interpretation and comparability of the study results. A virtual environment, while it may not yet be fully optimized, is utilized to ensure the realism of the training activities. The duration of the training sessions has been designed to fit within a regular physical therapy treatment session, to promote the potential for incorporating this type of training in the usual care process of patients in the future. While the sample in this study will be controlled by inclusion and exclusion criteria to ensure that an at-risk sample is included, we decided not to include subjects based on a specific disease or disorder, to promote the external validity of the results. A 6 months’ follow-up of daily-life falls incidence and a 2-year follow-up of injurious falls have been included to measure the effect of this intervention on subjects’ daily life and their long-term risk of sustaining recurrent injurious falls in addition to the balance measured in the lab.

### Weaknesses

The main weakness of this study is that it is not powered for all included outcome measures. The power calculation was based on the main outcome, i.e. balance control measured with the mini-BESTest, while for older adults their real-life falls incidence may be more essential. However, powering the study for this outcome is not possible, as the required sample size would not be achievable due to practical (time, resources) constraints. Secondly, the choice to include a broad spectrum of perturbations in the training protocol is based on our conclusions from the literature. This may be the right approach to ensure that each subject is prepared for multiple types and directions of perturbation, but it may also have a negative effect because the training dose of each individual perturbation type is lower. The results of this study will indicate whether this was a good approach. Finally, a disadvantage of the intervention is that it is device-dependent and only individual training is possible. Even though efforts are made to improve the external validity of the study findings, the feasibility of applying this intervention in usual care will have to be investigated in future studies.

## Supplementary Information

**Additional file 1.**

## Data Availability

Not applicable.

## Abbreviations

PBT: Perturbation-based balance training
RCT: Randomized controlled trial
TFA: Theoretical framework of acceptability
MUMC+: Maastricht University Medical Center+
CAREN: Computer Assisted Rehabilitation Environment
Mini-BESTest: Mini Balance Evaluation Systems Test
FES-I: Falls Efficacy Scale International
EPR: Electronic patient record
SPIRIT: The Standard Protocol Items: Recommendations for Interventional Trials
NRS: Numeric Rating Scale
MAR: Missingness at random
METC: Medical Ethics Committee
